# Impact of the anodization time on the photocatalytic activity of TiO_2_ nanotubes

**DOI:** 10.3762/bjnano.9.244

**Published:** 2018-10-04

**Authors:** Jesús A Díaz-Real, Geyla C Dubed-Bandomo, Juan Galindo-de-la-Rosa, Luis G Arriaga, Janet Ledesma-García, Nicolas Alonso-Vante

**Affiliations:** 1 Centro de Investigación y Desarrollo Tecnológico en Electroquímica, 76703, Querétaro, México; 2 IC2MP, UMR-CNRS 7285, Université de Poitiers, 4 rue Michel Brunet, F-86073 Poitiers, France; 3 Facultad de Ingeniería, División de Investigación y Posgrado, Universidad Autónoma de Querétaro, Centro Universitario Cerro de las Campanas, Querétaro, Qro., C.P. 76010, Mexico; 4 The University of British Columbia, Clean Energy Research Centre, 6250 Applied Science Lane, Vancouver, British Columbia, V6T 1Z4, Canada

**Keywords:** fluorine doping, nanotubes, photocatalytic activity, photoelectrochemistry, titanium(IV) oxide (TiO_2_)

## Abstract

Titanium oxide nanotubes (TNTs) were anodically grown in ethylene glycol electrolyte. The influence of the anodization time on their physicochemical and photoelectrochemical properties was evaluated. Concomitant with the anodization time, the NT length, fluorine content, and capacitance of the space charge region increased, affecting the opto-electronic properties (bandgap, bathochromic shift, band-edge position) and surface hydrophilicity of TiO_2_ NTs. These properties are at the origin of the photocatalytic activity (PCA), as proved with the photooxidation of methylene blue.

## Introduction

TiO_2_ started to attract great interest after Fujishima and Honda reported [1] on its photoelectrochemical (PEC) properties in 1972. Numerous features such as excellent chemical stability, photo-corrosion resistance, low cost, and low toxicity make TiO_2_ a material suitable for energy production and environmental applications, such as advanced oxidation processes for the decomposition of organic pollutants in water [2–3]. However, the material generates charge carriers (electron–hole pairs) only under UV irradiation limiting the overall performance. This fact motivated the research on this material in order to increase the surface area and/or shift its light absorption toward the visible spectral region. Many reports are devoted to novel synthesis methods resulting in a wide range of morphologies, such as mesoporous structures [4], microtubes [5], microdendrites, nanoparticles [6–8], nanorods [9–10], nanotubes [11–13], nanowires [14], and nanosheets [15–16]. Standing out from the rest of the synthesis techniques, electrochemical anodization produces vertically oriented, well-ordered nanotubular arrays with high aspect ratio. TiO_2_ nanotubes (TNTs) produced in this way have been preferred by several groups since they are expected to exhibit better photocatalytic properties than nanoparticles, due to the short electron-diffusion path, high specific surface area, high mass-transport rate, and remarkable light-harvesting properties [17–18]. Generally, the electrochemical anodization process implies that the anodic polarization of a mechanically/chemically prepared Ti sheet induces the growth of TNTs using an etching agent (typically fluorine ions). Important contributions to the underlying mechanisms in the growth and morphological aspects of TNTs were given by Macak et al. [19] and Albu [20]. The simplicity of generating such highly organized structures resulted in a large effort to finely control their morphology [21–23]. More importantly, it has been recognized that several parameters of the anodization, such as electric field strength, water content in the electrolyte, concentration of fluorine ions and pH value, have a direct influence on the electronic properties of the TNTs [20].

Nevertheless, the modification procedures for the as-prepared TiO_2_ materials usually report the use of wet-chemical routes, ion implantation, and calcination under reducing atmospheres, among others approaches [4,24–25]. However, some of these methods have shown detrimental effects on the opto-electronic properties of TiO_2_. Su et al. [26] observed that a water-based electrolyte containing NH_4_F induced a co-doping with F and N in the TNTs. Their study suggested that a combination of applied potential and annealing temperature were responsible for the high photocatalytic activity (PCA) of their materials in the oxidation of methyl orange. In a different approach, Marien et al. observed a relation between the morphological features of TNTs and a higher yield in the degradation of Paraquat [27]. They proposed that the PCA of nanotubes is compromised by the length of these structures due to the diffusivity of the pollutant into the inner area of the TNTs. However, the physicochemical and the photoelectronic properties of the TNTs were not considered in their work. For example, the well-documented etching effect of the F^−^ ions was not considered.

Herein, we prepared TNTs by anodization in a typical ethylene glycol-based electrolyte and systematically studied the influence of the anodization time on the morphological, structural, surface-chemical, photo-electrical, and opto-electronic properties in order to explain their PCA in the degradation of methylene blue (MB).

## Results and Discussion

### SEM/EDS

**Figure 1:**
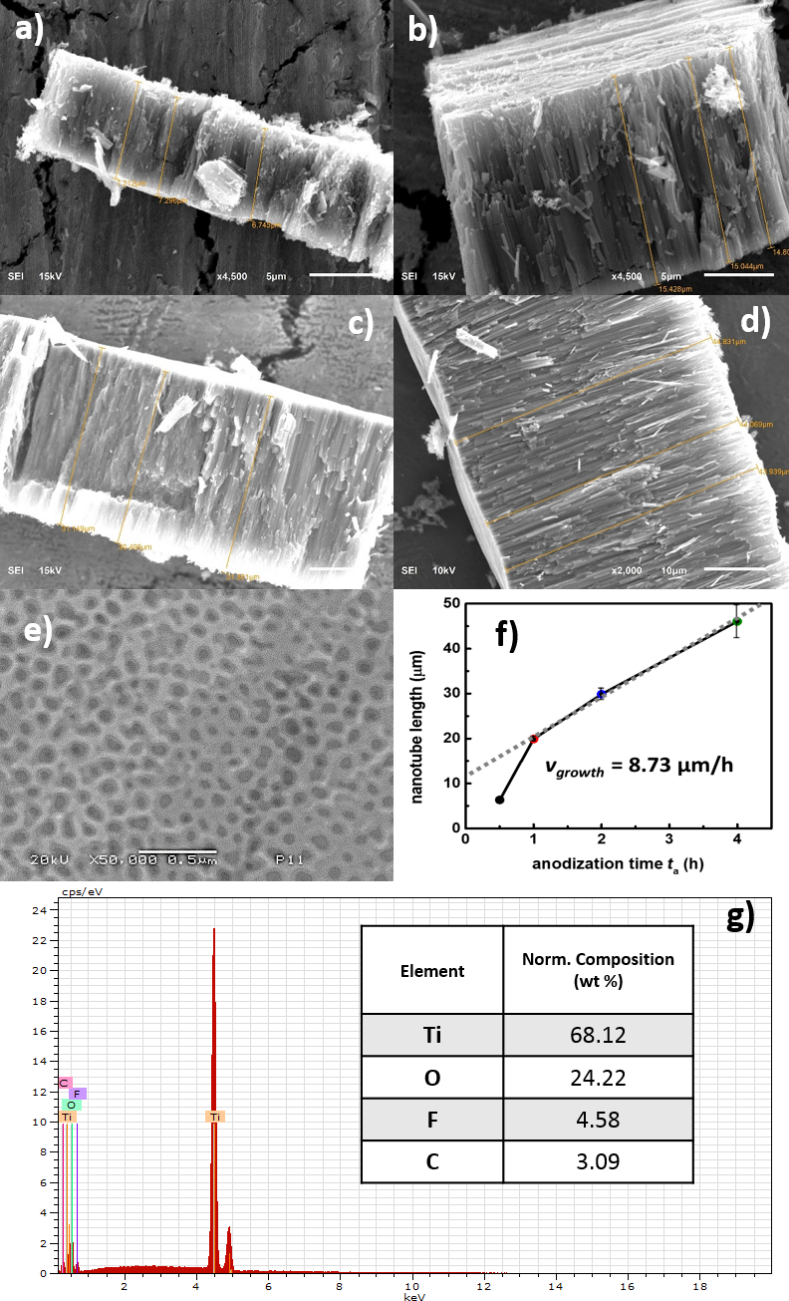
SEM images, a) top and b) cross section, of TNTs for each of the anodization times (*t*_a_): c) 0.5 h, d) 1 h, e) 2 h, and f) 4 h. g) EDS spectra of a TNT array after 0.5 h of anodization.

The morphology of the samples prepared for each anodization time, *t*_a_, was investigated via scanning electron microscopy (SEM), as presented in Figure 1. Well-defined, regular, nanotubular arrays are observed at the surface of these materials. It has been previously reported that *t*_a_ determines some of the geometrical characteristics of such nanostructures [20]. We determined the length of the nanotubes from the cross sections of these layers (detached from the metallic substrate), yielding 6.35, 19.9, 29.87 and 46.07 μm for *t*_a_ values of 0.5, 1.0, 2.0 and 4.0 h, respectively (Figure 1c–f). In our case, we observed a linear trend for the growth of these TNTs in terms of length after 1 h of anodization and we calculated an apparent growth rate of 8.73 μm/h (Figure 1f). The internal diameter of the nanotubes did not seem to change significantly, ranging from 70 to 79 nm, which is consistent with the observations reported in the literature [11,13,28]. For the elemental composition, a typical EDS spectrum for a sample with *t*_a_ = 0.5 h is presented in Figure 1g, where the presence of F and C are revealed. With increasing *t*_a_, the content of F in the TNTs varied within the range of 2.4 to 4.6%, while for C it was from 1.2 to 3.1%. Other authors have previously demonstrated that the carbon from the electrolyte disappears after heat treatment [20]. Since all the samples were annealed, these signals cannot correspond merely to the remaining electrolyte, suggesting that some of these elements have been incorporated into the structure of the TNTs. This idea is supported by the fact that atomic radii for O (48 pm) and F (42 pm) are similar enough to allow for the replacement of the former, effectively doping the material by creating oxygen vacancies and different energy states [29].

### XPS

**Figure 2:**
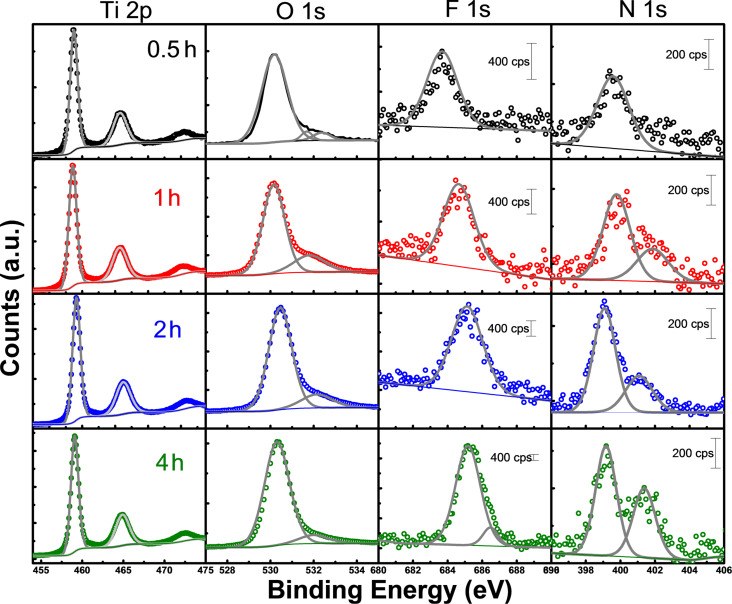
High-resolution XPS spectra for Ti 2p, O 1s, F 1s and N 1s obtained for the TNTs after each *t*_a_.

To characterize the surface chemistry, high-resolution XPS measurements were performed and the results are shown for Ti 2p, O 1s, F 1s and N 1s in Figure 2 and Table 1. All measurements were obtained after thermal annealing, for which the discussion of composition in terms of carbon is not considered. At first glance, the presence of fluorine can be observed which supports the idea of a fluorination. The proposed reaction process is ≡Ti–OH + F^−^ → ≡Ti–F + OH^−^ occurring through an inner-sphere ligand substitution reaction with hydroxy groups [30]. The F 1s spectra show an asymmetrical shape, arising from different interactions of the F atoms, with a major peak at a binding energy of 683.7 eV, which corresponds to the (≡Ti–F) surface species [31], or physically adsorbed F^−^ ions [32], supporting the proposed mechanism. With increasing *t*_a_, the peak becomes more intense and shifts to values of 685.2 eV, which have been associated with the formation of TiOF_2_ structures [33]. However, the spectrum of the 4 h sample shows the presence of a smaller peak at 686.5 eV, which is in the range of substitutional F replacing O atoms resulting in a structure of the type TiO_2−_*_x_*F*_x_* [24,32]. The intensity of the peak for F 1s showed a direct dependence with *t*_a_ and can be interpreted as a function of the exposure time of the TNTs to the F^−^ ions from the electrolyte (Figure S1a, Supporting Information File 1). It has been demonstrated by Albu [20] that a prolonged exposure of the TNTs to a fluorinated electrolyte leads to dissolution at the electrode/electrolyte interface. Fluorinated structures show physical modification (e.g., thinning of outer and inner diameter) via chemical etching through the fluoride ions dissolving the oxide (TiO_2_ + 6F^−^ + 4H^+^ → [TiF_6_]^2−^ + 2H_2_O). This change in the surface geometry is accompanied by a modification of the surface composition and is mirrored also in the O 1s spectra. The main peak (O 1s I, 530 eV) is usually attributed to Ti–O bonds, while the secondary one is attributed to surface hydroxy groups, Ti–OH, (O 1s II, 531.6 eV) and is often described as a characteristic of systems with enhanced photocatalytic properties [35]. An increased concentration of surface hydroxy groups is generally observed in doped TiO_2_ nanostructures, and these are indicated through the asymmetry of the O 1s spectra [32,36]. Ti–OH groups act as electron traps that not only improve the separation efficiency for electron–hole pairs, but also improve the generation of free radicals that are responsible for the degradation of organic molecules. As the *t*_a_ increased, the signal for surface OH groups was enhanced, suggesting a direct relationship with the fluorine content [34]. A third feature (O 1s III, 532 eV) was also present in the oxygen spectra and was ascribed to the presence of adsorbed water. A signal near 400 eV ascribed to N 1s was observed, which other authors associate with chemisorbed molecular N_2_. This signal becomes stronger as *t*_a_ increases, splitting in two peaks at 401.8 eV (NH_4_^+^ remaining from the electrolyte) and 399 eV. This last emission peak is within the range of signals connected to interstitial N, (O–N^•^–Ti), and has been associated with electron transitions from Ti^3+^/oxygen vacancy centers to interstitial N atoms [35,37–39]. These substitutional N atoms can also be associated with the enhanced signal in the OH group band (531.5 eV) [40–41]. These observations are relevant to our study since the presence of both F and N signals suggests a co-doping effect, as shown in Figure S1a (Supporting Information File 1). Moreover, the modification of the bandgap can be ascribed to the doping effect and will be discussed in the following sections.

**Table 1:** Binding energy (*E*_B_) values for the peaks of the high-resolution XPS spectra.

peak	*E*_B_ (eV)			
	0.5 h	1.0 h	2.0 h	4.0 h

Ti 2p_3/2_	459	458.9	459.3	459.2
Ti 2p_1/2_	464.6	464.6	464.9	464.9
O 1s I	530.2	530.2	530.5	530.4
O 1s II	531.6	531.8	532.2	532
O 1s III	532.4	—	—	—
F 1s I	683.7	684.5	685.2	685.2
F 1s II	—	—	—	686.5
N 1s I	399.5	399.9	399.4	399.2
N 1s II	—	401.8	401.9	401.4

### XRD

**Figure 3:**
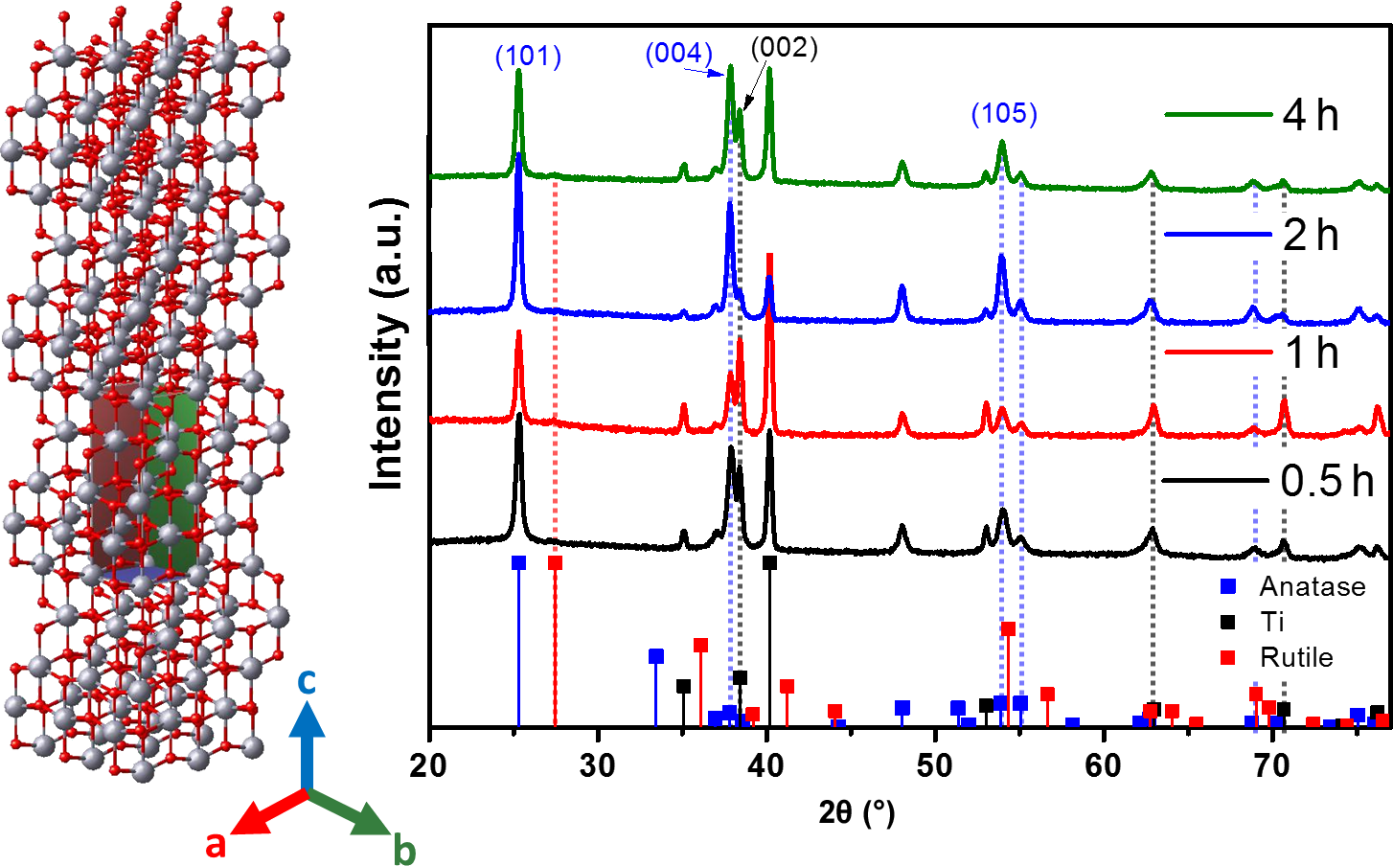
X-ray diffraction patterns of TNT grown at different *t*_a_.

The structure and crystallinity of the samples were studied by means of XRD and the diffractograms are presented in Figure 3. The Bragg–Brentano geometry was preferred for our work with thin films. After being thermally treated, all the samples exhibited the typical Bragg positions of anatase (PDF#21-1272) with its highest intensity peak at 25.3° for the (101) plane. However, an interesting feature of these samples is the signal at 2θ = 37.8°, corresponding to the (004) plane, which according to the reference XRD pattern has a relative intensity of 20% of the highest peak [42–43]. This peak exhibits an increasing relative intensity with higher *t*_a_, surpassing that of the (101) plane, and the ratios of the peak intensities for those planes are reported in Table 2. Acevedo-Peña et al. observed an enhancement of the thermal stability in anatase nanotubes with a preferred orientation along the [004] direction [44], accompanied by a higher photocatalytic performance in the degradation of an organic pollutant [45]. Lee et al. also reported an enhanced photoelectrochemical behavior in photovoltaic devices ascribed to the preferred crystalline orientation due to faster electron transport [46]. While the observed metallic Ti is attributed to the metal substrate, no significant contribution of the rutile phase was observed. The latter is also consistent with the observations of Dozzi et al., where even small amounts of F dopant atoms prevented the thermal transition from anatase to rutile, confirming their role in the crystalline stability [47–48]. The crystallite size was calculated by using Scherrer equation or Rietveld refinement, and the lattice parameters were estimated using the software TOPAS. The results are summarized in Table 2, where it was found using both methods that a higher *t*_a_ resulted in increasing crystallite size. It was found also that the lattice parameters have a small expansion with higher *t*_a_, particularly in the *c*-direction (Figure S1b, Supporting Information File 1) [49]. Finally, a unit cell was built with the aid of the software TOPAS depicting the growth orientation with an expanded representation of 3 × 3 × 4 cells as shown in Figure 3. Taking into account the XPS analyses, a higher concentration of F^−^ ions could be responsible for such changes in the lattice parameters as a result of the doping effect, which might be reflected in the electrical and optical properties and this is further discussed in the following sections.

**Table 2:** Summary of structural parameters estimated from XRD diffractograms.

*t*_a_ (h)	*a* (Å)	*c* (Å)	unit cell volume	crystallite size (nm)		(101)/(004)
				Scherrer	Rietveld	

0.5	3.7828	9.4830	135.6977	30.9	31.6	1.24
1	3.7814	9.4922	135.7288	34.6	36.6	1.46
2	3.7833	9.4965	135.9268	37.5	43.5	1.35
4	3.7841	9.4934	135.9399	34.5	42.7	0.97

### Raman spectroscopy

**Figure 4:**
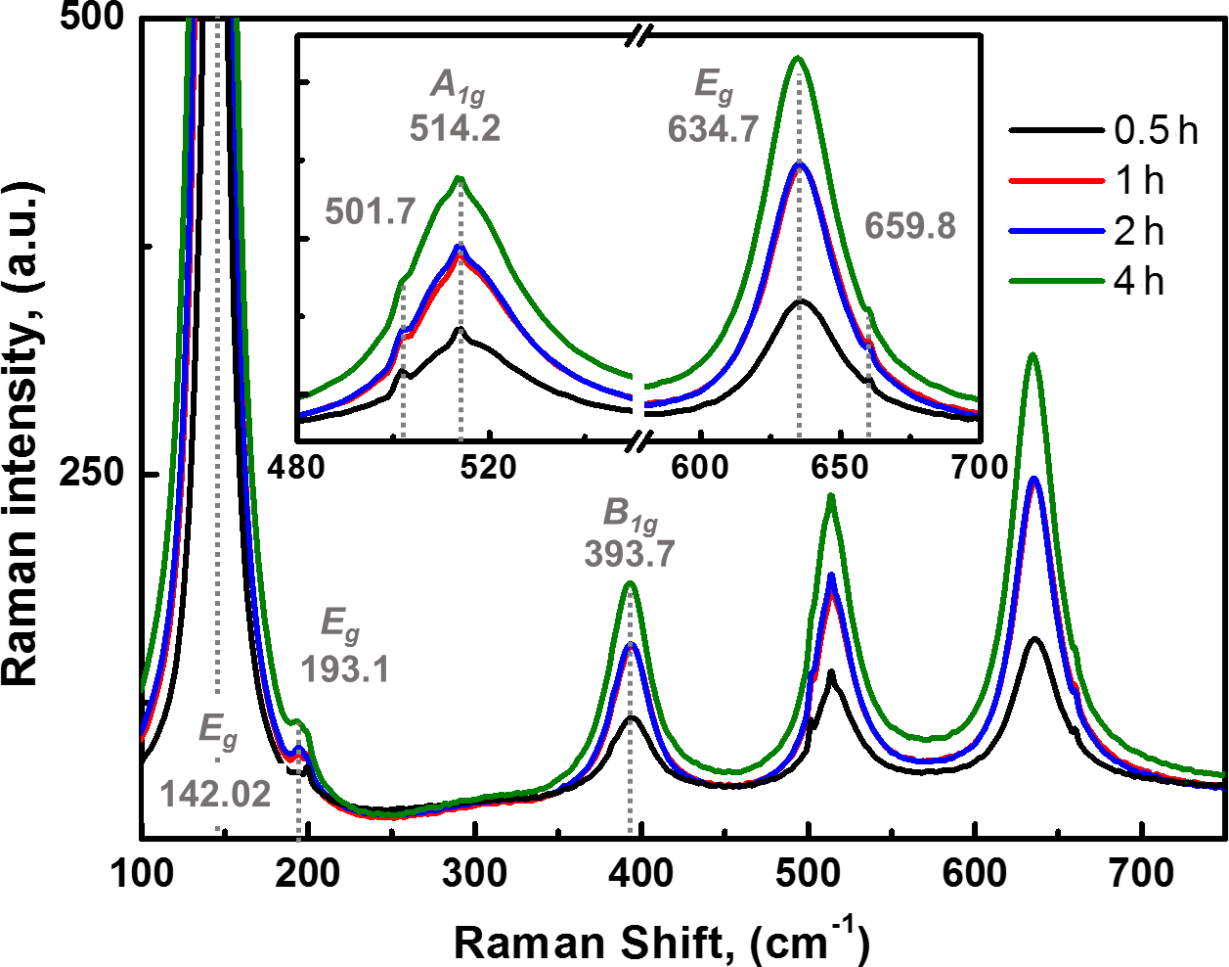
Raman spectra for TNT grown at different *t*_a_. Laser intensity: 2.5 mW/cm^2^.

Raman spectra obtained after annealing at 450 °C in air are shown in Figure 4. Anatase is the stable phase with bands at 193.1 (E_g_), 393.7 (B_1g_), 514.2 (A_1g_), and 634.7 cm^−1^ (E_g_), as well as a sharp and intense peak at 142 cm^−1^ (E_g_) assigned to anatase lattice vibrations [50]. No peaks of the rutile phase were observed in any of the samples. This is in agreement with the XRD data and other reports [51]. It is also noticeable that the intensity of all peaks increases with the anodization time without peak shifts. Two small peaks centered at 501 and 660 cm^−1^ were obtained after an instrumental artifact was canceled out by measuring a TiO_2_ control sample (Figure S2, Supporting Information File 1). The vibration at 660 cm^−1^ has been observed in titanate nanotubes and was attributed to Ti–O anatase vibrations; while the one at 500 cm^−1^ experimentally matched the theoretical approximations by Iliev et al. for the brookite phase [52]. Based on these results, it is reasonable to assume that the anodization time does not influence the crystalline phase and that the phase composition merely depends on the annealing conditions. An interesting remark from the literature comes from Hardcastle et al. [53] who observed that anodically grown TNTs after heat treatment between 400 and 500° C showed a 6.5-fold increase in the photocurrent density when the crystalline phase changed in proportion from 72% anatase (400 °C) to ca. 8% (500 °C). This observation is of interest since most of the available literature suggests that the most photoactive crystalline phase is anatase and that the rutile phase appears at higher temperatures (above 500 °C) [44,54–55].

### Electrochemistry and photoelectrochemistry

**Figure 5:**
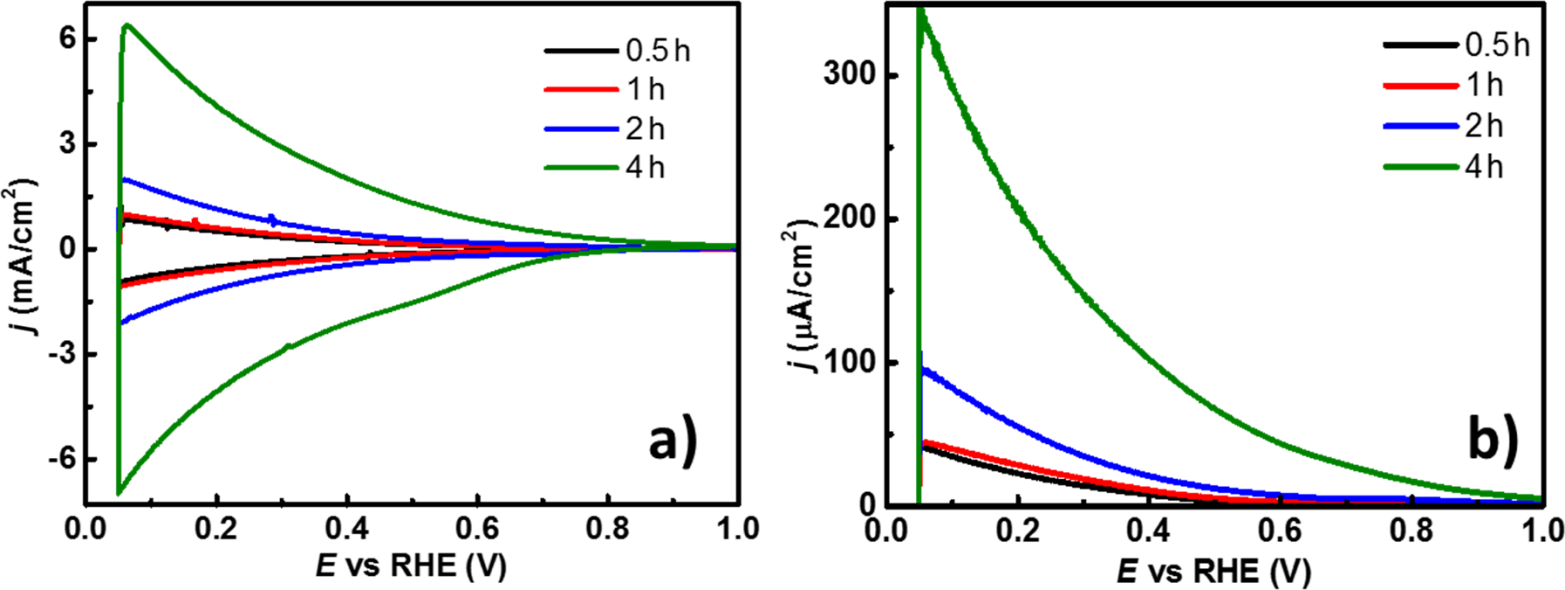
(a) Cyclic voltammograms (CV) at *v* = 50 mV/s; and (b) linear-sweep voltammograms (LSV) at *v* = 5 mV/s in the anodic direction for the TNTs grown at different *t*_a_, in 0.5 M H_2_SO_4_, in darkness in N_2_-saturated electrolyte.

A systematic approach was conducted for a better understanding of the above discussed properties. Initially, cyclic voltammograms (CVs) were recorded in darkness at a scan rate (*v*) of 50 mV/s within a potential window from 0.05 to 1.0 V/RHE and normalized to the geometrical area (Figure 5a). The scanning starts at open-circuit potential in the anodic direction where the current density, *j,* decreases at higher potential values, a characteristic for a passive layer [56–57]. After reaching the upper inversion potential and changing the scan direction, *j* increases towards lower potential values in a symmetrically inversed fashion, where *j* corresponds to the charging of the electrode (i.e., capacitive current). This quasi-reversible behavior is indicative of a stable surface. In the potential range from 0.05 to 0.6 V/RHE, |*j|* increases for samples with higher *t*_a_ and, since *j* is an extrinsic property, we attributed this to the different nanotube lengths and/or increase of the surface area. This observation is interesting when considering that the NT layers have an inherent superhydrophilic character allowing for easy electrolyte percolation [58]. However, the electrolyte percolation mechanism of the TNTs is complicated, and depends on the synthesis conditions, posttreatments and exposure to UV–vis light [59]. Hence, the enhanced current density could be related to an increased area of the TNTs, which depends directly on the increase of *t*_a_. To verify the degree of electrolyte percolation inside of the tubes and minimize the contribution of the charging current from the scan rate, linear-sweep voltammograms (LVS) were recorded at *v* = 5 mV/s in anodic direction from 0.05 to 1.0 V/RHE. A constant polarization at 0.05 V/RHE for 10 s was applied to the electrodes prior to running the LSV, as a pretreatment to minimize the initial charging spikes and to obtain a more accurate signal. The results are shown in Figure 5b and assessing those obtained from the CV measurements, provide support to the idea of *j* being a function of the nanotube length. The latter characteristic implies that the electrolyte is capable of permeating the external and/or internal part of the nanotubes, at least to some extent, which is highly desirable for catalytic applications. Other authors have stated several observations regarding the geometrical aspects of TNTs, where the nanotube length plays an important role in the overall performance of their specific systems [27,60–61].

**Figure 6:**
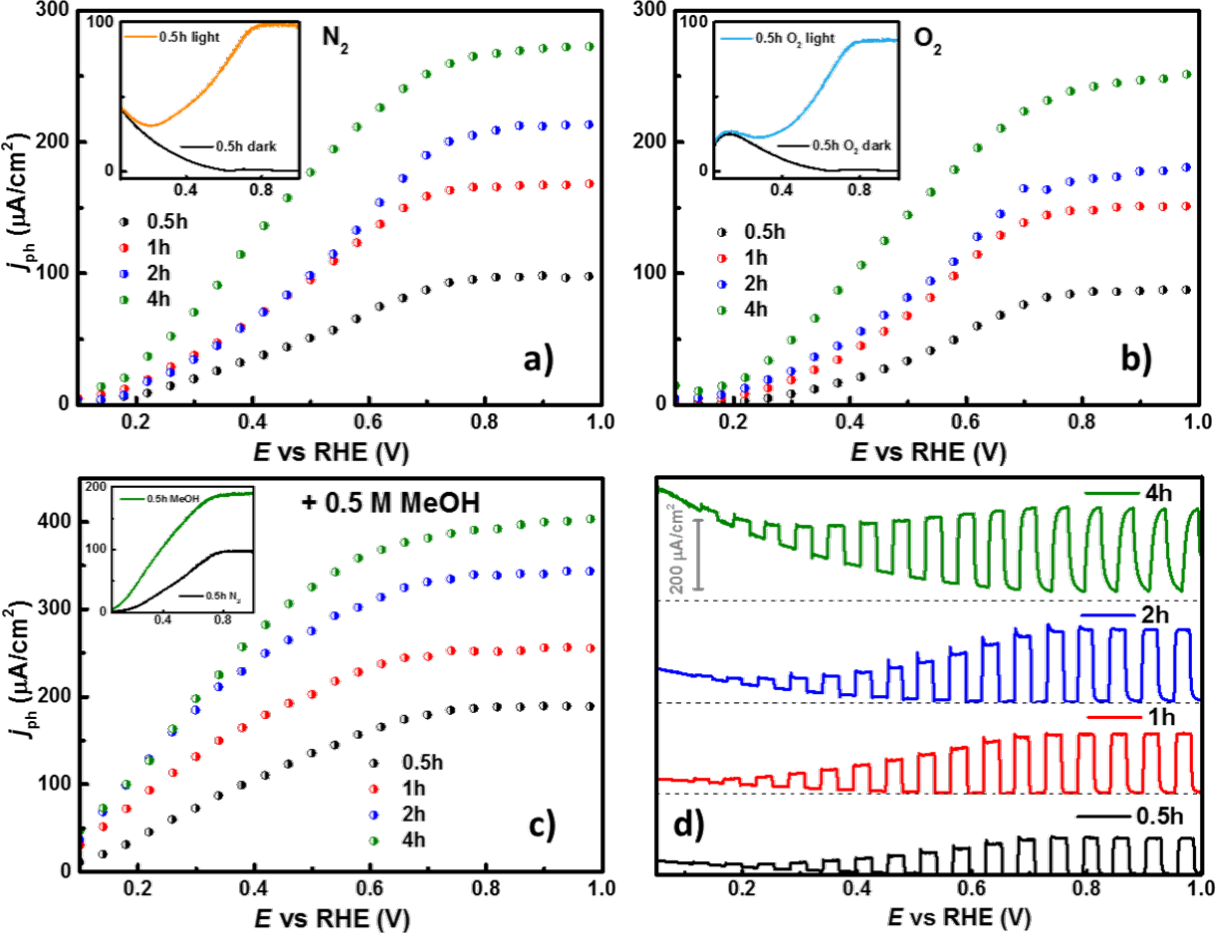
Extracted photo-current density, *j*_ph_, from potentiodynamic curves for the TNTs grown at different *t*_a_ in (a) N_2_-saturated electrolyte, (b) O_2_-saturated electrolyte, with (c) 0.5 M MeOH. (d) On–off photocurrent transients for the same samples in N_2_-saturated electrolyte. Conditions: 0.5 M H_2_SO_4_, *v* = 5mV/s, and irradiation power = 20 mW/cm^2^.

A similar approach was used to evaluate the PEC properties of the TNTs. For this purpose, the electrodes were illuminated in a cell with quartz window while performing LSV using the same pretreatment of 10 s polarization. The photocurrent, *j*_ph_, is then obtained as the difference between the current density under illumination (*j*_on_) and the current density under darkness (*j*_off_), (j_ph_ = *j*_on_ − *j*_off_). Figure 6a shows *j*_ph_ as a function of *t*_a_, displaying the same increasing trend. In the inset of the same figure, the difference between *j*_on_ and *j*_off_ for the sample with *t*_a_ = 0.5 h is shown. The photoinduced processes are dynamic phenomena that are affected by several factors such as the electrochemical parameters and the chemical environment. For example, high scan rates might lead to mass-transport limitations due to diffusion of the charge carriers within the bulk of the semiconductor. Subsequently, the electron percolation occurs in the TNTs, and crystalline defects and trap states (e.g., surface states) are among the factors that affect the electrical conductivity [62]. Thus, low scan rates are preferred in these experiments for more accurate measurements of the *j*_ph_. However, chemical species, such as O_2_, in the vicinity of the electrode are prone to scavenge the photogenerated electrons. For this reason, a comparative analysis was done with O_2_ saturated-electrolyte and the results are shown in Figure 6b. The maximum obtained value of *j*_ph_ decreased by ca. 20% compared to the measurement in N_2_-saturated electrolyte, revealing the scavenging action of dissolved oxygen. While electrons are generated and collected in the outer circuit, electron vacancies (holes, h^+^) are left behind in the valence band of the material, which have an elevated oxidation potential when molecular species are present. To evaluate the oxidative contribution of the photogenerated holes, methanol (MeOH) was added to the electrolyte. From Figure 6c it can be seen that the addition of 0.5 M MeOH increases the photocurrent *j*_ph_ up to 200% (Figure 6a). This refers to a phenomenon known as current-doubling in which a secondary electron is obtained from the oxidation of an (organic) molecule via surface holes when a semiconductor is excited with suitable photonic irradiation. However, the experimental results do not necessarily correspond to a two-fold increase in *j*_ph_, especially for longer TNTs (Figure S1c, Supporting Information File 1). This behavior might be due to structural defects, such as trap states, in good agreement with the discussion in previous sections (Table 1 and Table 2) or mass-transport limitations. The maximum of *j*_ph_ is obtained after reaching a certain potential, overcoming the recombination events, and characterized by a plateau. This potential was achieved near 0.7 V/RHE, while at lower applied electrode potential values a growing *j*_ph_ is observed that is hampered by electron–hole recombination processes. At even lower potentials it is possible to observe the onset potential, *E*_onset_. The latter is a specific energy condition where the photogenerated electrons are effectively separated due to the electric field at the semiconductor/electrolyte interface and where *j*_ph_ becomes observable. The importance of *E*_onset_ in these experiments relies on the fact that it practically merges at the flat-band potential, *E*_fb_, and allows us to roughly estimate the position of the conduction band in the electrochemical potential scale. From the LSV the *E*_onset_ of all samples seems to start as soon as the surface is irradiated. To discard an instrumental artifact, photocurrent transients were performed to discriminate the appearance of the *E*_onset_. The transients were performed by interrupting the irradiation for a period of 5 s with a programmable shutter coupled to the illumination during the LSV and the curves are contrasted in Figure 6d. For the electrode with *t*_a_ = 0.5 h, the transients revealed that in fact *E*_onset_ starts below 0.05 V/RHE, while for longer *t*_a_, *E*_onset_ shifts to higher values (Figure S1d, Supporting Information File 1). This observation has been associated with electron mobility and charge separation kinetics [63]. As the electrode is illuminated, some current spikes appear along with *j*_ph_*,* which are related to electron–hole recombination in surface states when the polarization potential of the electrode is not strong enough to prevent these events. At higher applied electrode potentials (around 0.7 V/RHE), these spikes disappear. For the electrode with *t*_a_ = 4 h, the spikes are significantly reduced even at smaller electrode potentials, which can be attributed to a possible faster filling of such energy states induced by the F or N atoms at the surface of the TNT. When the irradiation is interrupted, a fast decay of the photocurrent was observed due to recombination. If the potential is high enough to compete with the charge-carrier quenching, usually in the region where the current density achieves the plateau, the decay is prolonged and the influence of the nanotube length is distinguished. A longer decay was observed for longer nanotubes, which is also consistent with the idea that the electron conduction in these systems is governed by a diffusional gradient and, thus, the electron transport can be appreciated [64].

Similarly, with longer nanotubes the spikes not only disappear, the time to reach the maximum *j*_on_ also increases. While such behavior is still related to the length of the nanostructures, it could also be associated with an increased number of electron traps since the potential is high enough to overcome recombination [65]. The differences observed in the interfacial PEC behavior suggest that a link between the nanotube length and the chemical composition is established in agreement with the results obtained by XPS and XRD.

**Figure 7:**
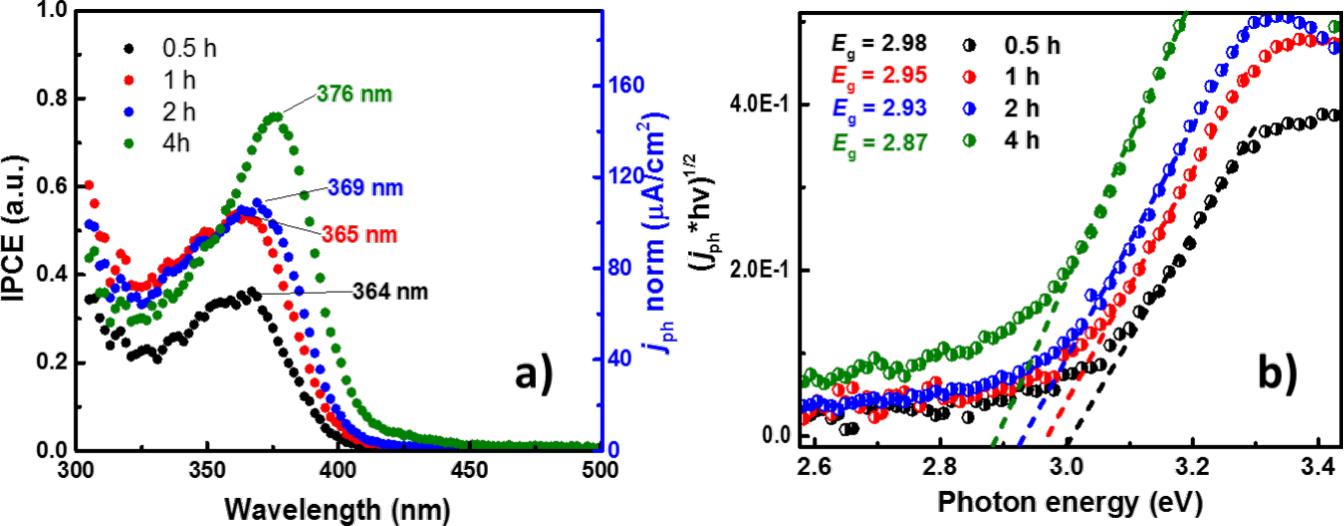
(a) Incident photon-to-current (IPCE) plot for each TNT. (b) Tauc plots for a direct electronic transition. Conditions: E = 1.0 V/RHE, 0.5 M H_2_SO_4_, 20 min N_2_ bubbling and irradiation power = 20 mW/cm^2^.

While the previous results were obtained from potentiodynamic experiments (LSV), a comparative analysis can be done by establishing a constant polarization to understand the sweep-rate dependence. Figure 7a presents the incident-to-photon conversion efficiency (IPCE) curves, which correlates the *j*_ph_ to the wavelength. A direct observation is the increase of the IPCE values for longer TNT in agreement with the LSV experiments. Moreover, a bathochromic shift occurs when *t*_a_ is varied. This shift not only displaces the maximum value of IPCE associated to a wavelength (λ_max_), but also to the absorption edge toward longer wavelengths [66]. This effect has also been observed for semiconductors where the crystalline structure has been changed by chemical doping. Other authors have reported an improved crystalline stability, attributed to the F dopant atoms inducing a higher UV absorption, while N dopant atoms were associated with the promotion of the red-shift [39,47]. Based on the results from XPS, XRD and photoelectrochemistry we suggest that the TNTs have undergone simultaneous doping with N and F. Nevertheless, some physical phenomena such as light scattering (from the top part of the TNTs) and oxygen vacancies could also create distortions in the IPCE spectra. Furthermore, the bandgap (*E*_g_) can be obtained from the Tauc plots, Figure 7b, plotting *j*_ph_ as a function of the wavelength (Equation 1):

[1]
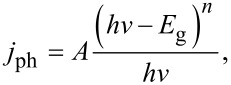


where A is a constant, and *n* is a parameter for direct (*n* = 0.5) or indirect (*n* = 2) optical transitions. For our study, an indirect optical transition was assumed (*n* = 2) since it is commonly accepted as the main process governing the nanocrystalline electrodes [67–68]. Then, the intersection between the extrapolated slope of the Tauc curve with the energy axis corresponds to *E*_g_ for the sample. The *E*_g_ values obtained for TNTs are lower than those associated with anatase (3.2 eV) [69]. Longer times *t*_a_ decrease the bandgap (Figure S1e, Supporting Information File 1), supporting our hypothesis of a doping effect originating from an extended anodization time (Figure S3a, Supporting Information File 1).

**Figure 8:**
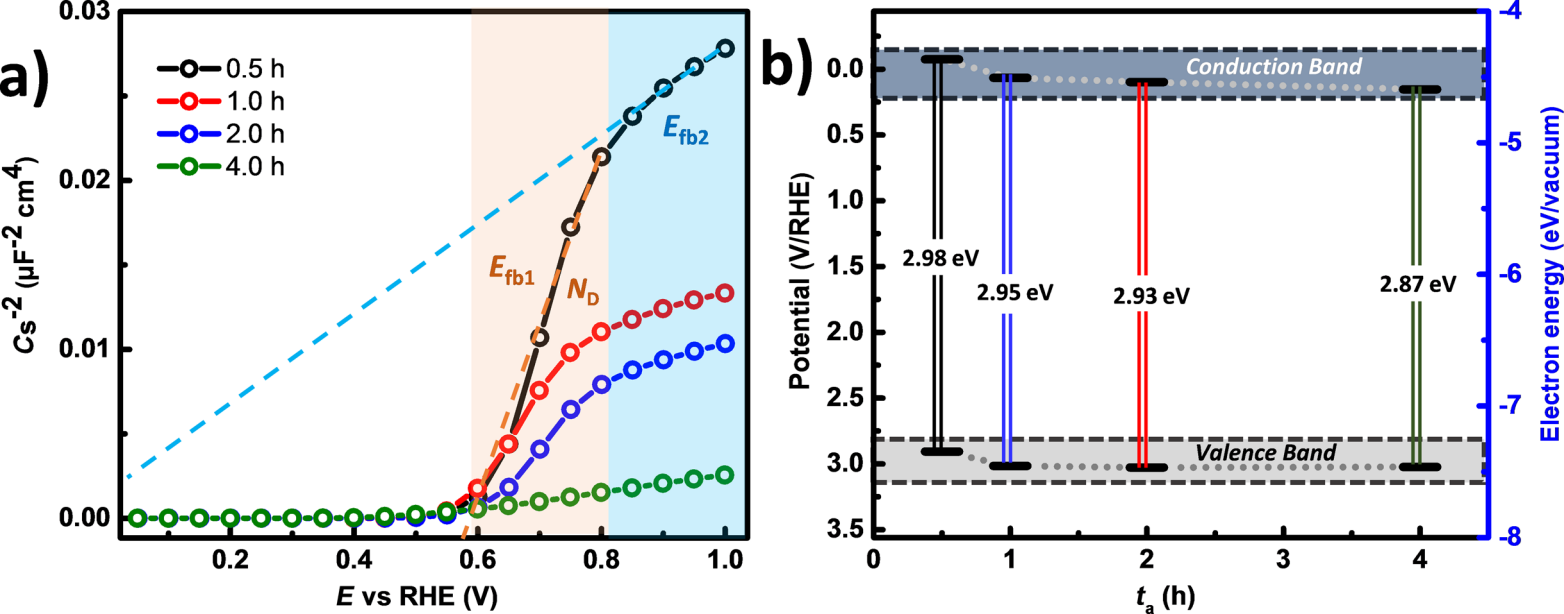
a) Mott–Schottky plots recorded at *f* = 400 Hz in 0.5 M H_2_SO_4_. The electrode potential range was from −0.2 to 1.0 V vs SCE with a potential step of 50 mV and 2 min of stabilization time. Table 3 shows the dopant number concentration (*N*_D_) calculated by using Equation 2. b) Schematic for the band diagram estimated from the measurements.

As it was stated in previous sections, a relation between the capacitive processes seems to be associated with the length of the TNTs. Consequently, electrochemical impedance spectroscopy was performed to access the capacitance of the space-charge region and then plot the Mott–Schottky (M–S) curves. Figure 8a shows the curves obtained for the samples, where the capacitance increases its value with decreasing potentials. From the slope, the number of dopant atoms can be obtained using the Mott–Schottky equation (Equation 2):

[2]
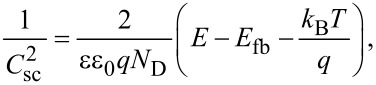


where *C*_sc_ is the capacitance of the space-charge region, *N*_D_ is the density of dopant atoms (cm^−3^), *E*_fb_ is the flat-band potential, *q* is the elementary charge (1.602 × 10^−19^ C), ε_0_ is the vacuum permittivity (8.85 × 10^−14^ F·cm^−1^), and ε (42, [70]) is the dielectric constant of the material. From the CV curves, an increase in the capacitive current was observed towards lower potential values, which is typical for a transition to the accumulation region of n-type semiconductors. The calculated values for *E*_g_ and *N*_D_ are summarized in Table 3, and compared to the data obtained by the PEC technique. In the literature, a wide range of values for *E*_fb_ have been reported and it has been found that this parameter can be affected by several properties of the material (crystallinity, doping level or synthesis conditions) as well as the chemical environment (ionic force, cation type or pH value). Numerous publications discuss the deviations from the linear behavior of the slope in the M–S curves. It has been proposed by Dean and Stimming [71] that this behavior arises from a distribution of energy states and the same authors reported an equation to account for such a dispersion. In an attempt to obtain a frequency-independent capacitance, some authors [72–75] have proposed the use of high frequencies, typically 10 kHz, to deal with this phenomenon. However, this criterion fails when porous substrates are evaluated since the ac perturbation cannot reach the interior of the pores [76]. In a different approach, Muñoz [77] demonstrated that the value for *E*_fb_ can sensitively change from a compact layer to a nanotubular material due to the distribution of states that depends in turn on the synthesis conditions. The distribution of these states will not be uniform along the length and the wall thickness of the TNTs due to etching during the growth process. In his work, he chose *f* = 3 Hz to avoid interferences from the double-layer capacitance. Considering the amorphous-semiconductor theory [78] in the interpretation of the M–S plots of Di Quarto et al. [79], the existence of several of bandgap states, leading to different *E*_fb_ values was assessed [77]. Thence, care should be taken when selecting the frequency for the M–S analysis. The criteria established by Gelderman et al. (∂ log Z″/∂ log *f* = −1 and constant Z′) [80] is fulfilled at *f* = 400 Hz, in the potential range from 0.8 to 1.0 V. Based on Muñoz’ arguments, it is reasonable to consider the dispersion of intra-bandgap states leading to the deviation of the M–S curves. For this reason, we estimated the *E*_fb_ in two ways 1) through extrapolation of the slope where *N*_D_ was calculated (*E*_fb1_), and 2) from the region of 0.8 to 1.0 V (*E*_fb2_). The latter represents an estimation of the *E*_fb_ value at the bottom of the tubes and agrees with those expected for short tubes, while the *E*_fb1_ agrees with those values expected for long tubes (0.5 V < *E*_fb_ < 0.6 V) [77]. The values of *E*_fb2_ are in agreement with the *E*_onset_ (from LSV), supporting the idea that electron–hole pairs occur initially at the bottom of the tubes via an electron diffusion mechanism.

**Table 3:** Experimental and calculated values from PEC measurements for TNTs as a function of *t*_a_.

*t*_a_ (h)	*E*_onset_ (V/RHE)^a^	*E*_fb1_ (V/RHE)^b^	*E*_fb2_ (V/RHE)^b^	*E*_g_ (eV)^c^	*N*_D_ (cm^−3^)^b^

0.5	0.05	0.57	−0.074	2.98	3.16 × 10^19^
1	0.076	0.53	0.066	2.95	6.15 × 10^19^
2	0.094	0.56	0.098	2.93	8.76 × 10^19^
4	0.12	0.52	0.154	2.87	7.17 × 10^20^

^a^LSV/transients, ^b^Mott–Schottky plots, ^c^Tauc plots.

For the case of *N*_D_, an increase with longer anodization time was obtained and is in agreement with our observations in the XPS measurements (Figure S1f and Figure S3b, Supporting Information File 1). The data demonstrate good agreement and corroborate the relations between the optical and electrical parameters and *t*_a_. Taking into account the information from these experiments, a scheme for the energy band positions of the TNTs can be generated (Figure 8b). Both valence and conduction band experienced a shift toward higher energies as a consequence of the inherent doping during the anodization process. A similar behavior has been observed when commercial [31], and prepared samples [32] where modified during their synthesis in a fluoride-rich environment leading to a red-shift in the absorption edge. The energy levels of conduction and valence band show a similar trend, moving towards more positive potentials (in the electrochemical scale). Since the conduction band was assumed to merge with *E*_fb_, the calculations for the valence band value were carried out only in terms of the energy gap of the material. This resulted in a more homogeneous value for the upper edge of the valence band for samples with *t*_a_ = 1.0, 2.0 and 4.0 h. The latter observation might suggest that the photogenerated holes for such samples might have higher oxidation power for photocatalytic applications.

### Photocatalytic activity of the TNTs

**Figure 9:**
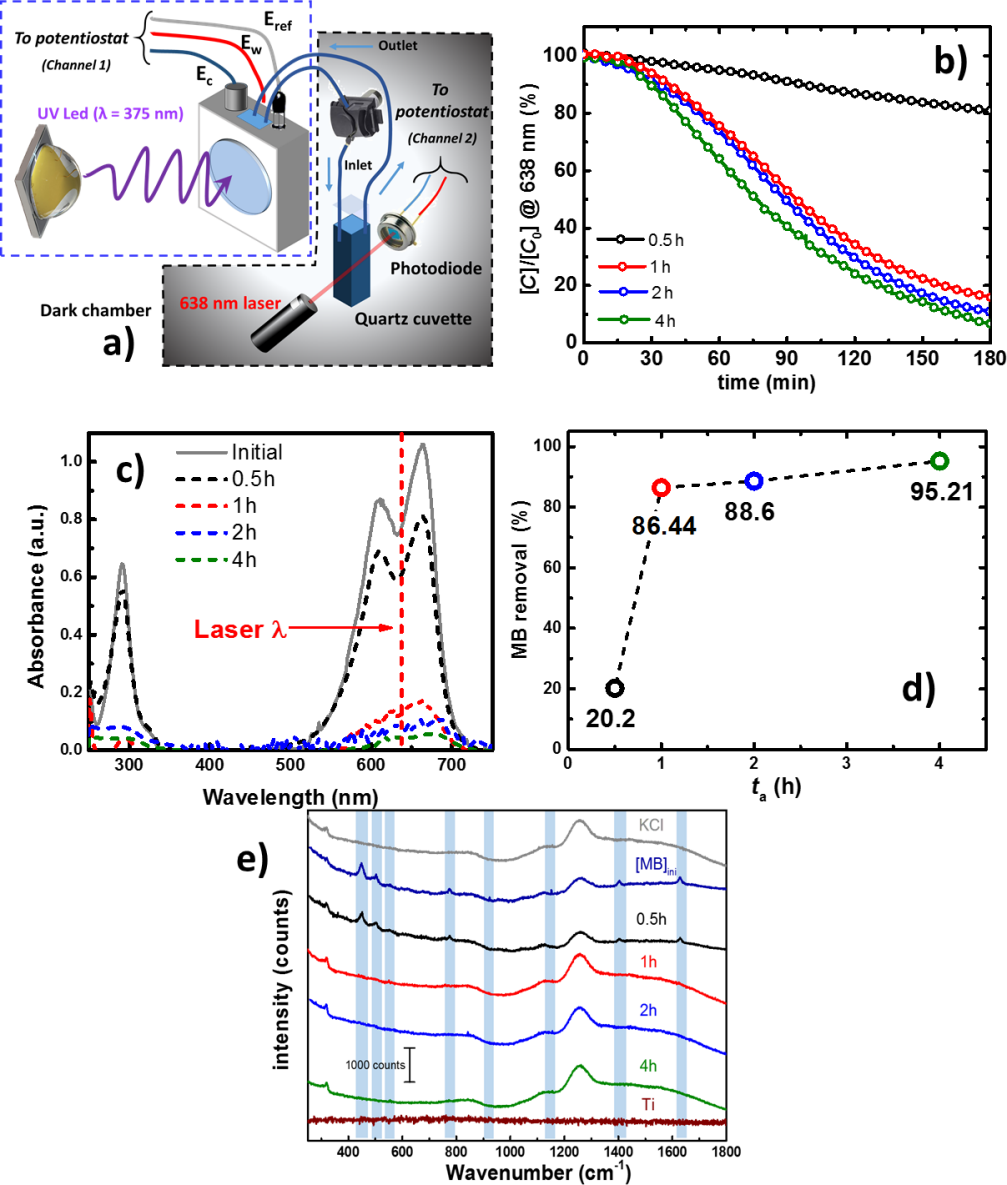
a) Schematics of the experimental setup used for the PEC degradation of MB. b) UV–vis spectra for the initial MB solution and after 180 min of treatment. c) Relative MB concentration followed in situ with a 638 nm laser. d) Removal percentage of MB evaluated from the UV–vis spectra. e) Raman spectra of the solution samples after 180 min of treatment. Raman conditions: laser 785 nm, slit 100 μm.

In order to investigate the PCA of the samples, the degradation of methylene blue (MB) was studied under UV irradiation. Ions are found in the waste waters from the textile industry resulting from the dyeing and bleaching processes, thus allowing the use of an electrooxidation process for the degradation of such components. Chlorides are often present in these waste waters [81–84], and for that reason a dissolution of 0.5 M KCl was chosen as the electrolyte. The electrolyte was not purged with N_2_ or O_2_ prior to or during the experiment. Figure 9a depicts the photoelectrocatalytic system used to perform the experiments. Briefly, a 20 mg/mL MB solution in the electrolyte was irradiated in a three-electrode electrochemical cell with a quartz window. An electrolyte/electrode (EE) configuration was used with a distance between the working electrode and the quartz cell (optical pass) of 1 cm. The MB dissolution was recirculated using a peristaltic pump to a standard quartz cuvette where the absorbance at 638 nm was measured in situ. In a typical experiment, a fixed ratio of 12.5:1 (volume/area) was used for all the samples while a potential bias of 1 V/(Ag|AgCl) was applied. In Figure 9b, the relative concentration of MB is presented and the results show that the oxidation proceeds at a low rate for the first 20 min. After this, a marked change in reaction rate is observed indicating a change in the reaction kinetics. The first stage is associated with an adsorption process that takes place independently of the potential bias or light irradiation [35,85]. It is important to underline that the understanding of this process is outside the scope of this study. After the initial phase, by the faster decay is associated with the heterogeneous photocatalytic processes explained by a Langmuir–Hinshelwood model [86]. The oxidation mechanism for organic compounds with irradiated semiconductors has been proposed to proceed via photogenerated holes at the surface of the electrode [87–88]. The cyclic voltammograms of TNTs with *t*_a_ = 0.5 h (Figure S4, Supporting Information File 1), present an anodic (−0.08 V/Ag|AgCl) and a cathodic (−0.21 V/Ag|AgCl) peak associated to MB. These peaks become absent in the presence of light, supporting the idea of the oxidation of MB by holes. In the presence of MB, *j*_ph_ decreases due to its light absorption and a lower *I*_0_ reaches the surface of the electrode. At 1.0 V/Ag|AgCl, *j*_ph_ is maximized and recombination is suppressed, which provides the conditions for efficient photoelectrochemical oxidation of MB. UV–vis spectroscopy was used to evaluate the effect of the treatment and the results are presented in Figure 9c. The couple of peaks with an absorption maximum centered at 663 nm, for the fresh solution, presents a slight decrease when treated with the electrode produced with *t*_a_ = 0.5 h, while for the rest of the electrodes the decrease is more significant. In the same figure, a dashed line shows the wavelength at which the in situ measurement was performed. The integrated signal from the UV–vis spectra is presented in Figure 9d to assess the extent of the photodegradation. From these results, it can be seen that TNTs generated with *t*_a_ ≥ 1 h exhibit superior performance, with a higher oxidation rate for higher *t*_a_. This trend in MB removal yield was found to be similar to that in the growth rate of the TNTs (Figure 1f), and the band-energy shift (Figure 8b). Furthermore, the charge that passed through the electrodes during the oxidation process was integrated and plotted as a function of *t*_a_ (Figure S5, Supporting Information File 1). A very similar trend to that observed in Figure 9d was found, supporting the idea that MB oxidation occurs through holes in the surface of the TNTs. Along with the increase in the length of TNTs, the higher content of surface hydroxy groups [89] for samples with *t*_a_ ≥ 1 h shows to have a beneficial effect for the degradation of MB, which could be also facilitated by an anchoring mechanism to a more hydrophilic TiO_2_ surface [90].

Further information on the degradation of MB was obtained with Raman spectroscopy. Figure 9e shows the spectra recorded for the solutions at the end of the treatment depositing a drop on a Ti sheet. The spectra of the electrolyte and Ti plate were considered as well, to cancel out their corresponding bands that are still visible in the solution spectra. The characteristic assignments for MB were within the range of 1800–250 cm^−1^ and have the following interpretation: C–N–C skeletal deformation (448 and 501 cm^−1^), C–S–C skeletal deformation (596 cm^−1^) in-plane bending mode C–H (770, 951 and 1154 cm^−1^), in-plane ring-deformation mode C–H (1302 cm^−1^), asymmetrical stretching C–C (1502 cm^−1^), and two prominent peaks for C–N symmetrical stretching (1394 cm^−1^) and C–C ring stretching (1623 cm^−1^) [91–92]. The vibrational bands observed in the recorded spectra agree with those values reported in the literature. The band found at 923 cm^−1^ assigned to in-plane bending of the C–H bonds, showed a slight shift from the reported value (951 cm^−1^). The signals at 448, 501, 596 and 1394 cm^−1^ can be attributed to the cleavage of the thiazine ring, since a considerable reduction in the band intensity assigned to C–N–C and C–S–C skeletal was observed for samples with *t*_a_ ≥ 1 h. Degradation of the aromatic rings was assessed based on the decreased vibrational band at 1623 cm^−1^, or on the breakdown of the chromophore feature.

From the results we understand that, although a trend is observed for the MB removal with increasing *t*_a_, it does not correspond in the same proportion to what it is obtained through the PEC experiments. In part, this has to be associated to the fact that the PCA was assessed in a continuous-flow regime, while the PEC experiments were not. This would suggest that once the MB has saturated the surface of the electrode (nearly 20 min after the experiment started), the real kinetics of the reaction were observed. At this point, the oxidation of MB on the TNTs does not correspond directly to their length, but more to the amount of surface hydroxy groups (induced by the fluorination and N dopant atoms) observed by XPS, which is related to the anchoring process. Thus, while the total exposed area (inner or outer walls of the tubes) remains important for the MB absorption step, the hydrophilicity of the surface is what it leads to the difference in the aforementioned trends, demonstrating the determining the influence of *t*_a_ on the morphological, surface chemical and opto-electronic properties of the TNTs.

## Conclusion

The impact of the anodization time, *t*_a_, on TNTs was studied. The TNTs were evaluated via different physico-chemical and electrochemical techniques and their photocatalytic activity in the degradation of MB was assessed. The only parameter that differed during the synthesis of the materials was the anodization time (*t*_a_). Longer TNTs were obtained for longer *t*_a_. A linear trend for the growth rate was observed for samples with *t*_a_ ≥ 1.0 h. Changes in the surface chemistry were confirmed by XPS measurements, indicating F and N doping. This was explained in terms of the exposition time during the anodization process since higher F contents were found for longer *t*_a_. The crystalline structure presented a preferential growth in the [004] direction. The presence of F in the TNTs was associated with the preservation of the anatase phase. The photochemical and electrochemical properties of the TNTs were studied, exhibiting increasing values of *j*_ph_ for longer nanotubes. Along with an increase in the current density for the accumulation region, the percolation of the electrolyte in the nanostructures was confirmed. The kinetics of the filling of trap states and the decay of the *j*_ph_ became sluggish when the length of the nanotubes increased, as supported by the voltammograms. A red-shift in the absorption edge of the TNTs was observed from Tauc curves attributed to F and N dopant atoms. The values of *N*_D_ obtained by the Mott–Schottky plots confirmed the doping of the material. Finally, the photocatalytic activity was assessed by measuring the degradation of MB. A higher degradation performance was obtained from the samples with *t*_a_ ≥ 1.0 h. The enhanced response was attributed to the energetic level of the photogenerated holes at the valence band, the energy level of which was assessed by various techniques, and the higher content of surface hydroxy groups induced by the fluorination of the TNTs.

## Experimental

### Preparation of TiO_2_-NT electrodes

TiO_2_ nanotubes were fabricated by electrochemical anodic oxidation of Ti foils (99% purity, 0.127 mm, Alfa-Aesar) [93]. Briefly, the foils were pre-treated with sandpaper, #600, #1200 and #2000 successively, thereafter immersed in ethanol (Sigma-Aldrich), placed in ultrasonic bath, for 15 min, and dried under N_2_ flow. An ethylene glycol-based solution with a concentration of 0.1M NH_4_F (96% purity, Alfa-Aesar) and 2% w/w deionized water was used as electrolyte. A Pt foil (99.9% purity, Alfa-Aesar) was used as counter-electrode and was placed at a constant distance of 2.5 cm from the working electrode. The electrode area (0.67 cm^2^) exposed to the electrolyte was kept constant. The anodization times used were 0.5, 1, 2, and 4 h with a voltage step of 60 V (BK Precision, model 9184). After the electrochemical anodization, the samples were immersed in ethanol to remove any remaining electrolyte and finally were dried under N_2_ flow. The as-prepared samples were annealed in a furnace for a period of 2 h at 450 °C with a ramp of 20 °C/min in air. The samples were let to cool down to room temperature under air and stored.

### Structural characterization

Surface composition and morphology of the samples were investigated by SEM (JEOL, model JSM-6510LV) equipped with energy-dispersive spectroscopy detector (EDS, Bruker XFlash 6I10). Crystallinity information was obtained from X-ray diffractograms performed on a Bruker AXS (model D8 Advance) diffractometer, using Cu Kα radiation and Bragg–Brentano geometry. Raman spectroscopy was also performed for all samples with a Micro-Raman Spectrometer (Thermo Nicolet, model DXR) equipped with a 780 nm laser and a 12× optical microscope. Spectra were measured at a laser power of 2.5 mW/cm^2^ and a slit width of 25 μm. X-ray photoelectron spectroscopy (XPS) was used to complement the information of the surface chemistry with a Thermo Scientific instrument (model K-Alpha + surface analysis).

### Electrochemical and photo-electrochemical characterization

The electrochemical measurements were performed at 25 °C in a three-electrode cell equipped with a quartz window using a SP-300 Bio-logic potentiostat. A glassy carbon and a reversible hydrogen electrode (RHE) were used as counter and reference electrode, respectively. The photoelectrodes were fabricated with an electrical contact of a Cu wire and colloidal-Ag paste (Radiospares 496-256). The TiO_2_-NT area was exposed by isolating with epoxy glue (3M, DP-190). Cyclic voltammetry (CV), in N_2_-saturated 0.5 M H_2_SO_4_ electrolyte was performed to clean the electrode surface from 0.05 to 1 V/NHE at a scan rate of 100 mV/s. 15 cycles were necessary to obtain a constant current density. Linear sweep voltammograms (LSVs) were recorded at 5 mV/s under N_2_ and O_2_ atmosphere. Light source was a Xe lamp (Spectral products, ASB-XE-175) coupled to a motorized monochromator (Horiba Jobin Yvon 0106-07-07) for photo-electrochemical characterizations. A UV hot-mirror filter (Edmund Optics F45-066) was used to prevent the heating of the electrolyte and to allow light with an irradiance power of 20 mW/cm^2^ to pass. Photocurrent transient curves were obtained by blocking the light for periods of 5 s with an automated shutter (Lambda SC Smart Shutter, LB-SC). Action spectra were obtained by applying a potential bias of 1.0 V/(Ag|AgCl) and carrying out a wavelength scan from 305 to 600 nm normalized to the lamp spectrum. Tauc plots were calculated using these data. Mott–Schottky curves were obtained in the same electrolyte, allowing the electrode to reach steady-current conditions prior to each measurement.

### Photoelectrochemical degradation of methylene blue

The degradation of MB was performed in a three-electrode electrochemical cell with a quartz window allowing for the illumination of the working electrode with a specific area of 1 cm^2^. Graphite and Ag|AgCl electrodes were used as counter and reference electrode, respectively, in a 0.5 M KCl electrolyte. A 375 nm UV-LED was used as illumination source with an effective irradiation power (*I*_0_) of 22.4 mW/cm^2^ at the working electrode distance, while recording the current under a potentiostatic bias of 1 V/Ag|AgCl for a period of 180 min. Simultaneously, the kinetics of the degradation of MB were followed using the transmittance of a 638 nm red laser and the solution measured with a photodiode at fixed distances. The evaluated solution was fed into a quartz cuvette with a peristaltic pump to recirculate it to the PEC cell using a volume of 12.5 mL. At the end of the PEC treatment, the absorbance of the solution was analyzed using an UV–vis spectrometer (Optizen, model Pop). Raman spectra were recorded for these solutions with a Horiba Jobim-Yvon instrument (model Xplora ONE) equipped with a CCD detector and holographic grating of 1200 grooves/mm.

## Supporting Information

File 1 Additional experimental data.
