# Supplementary material for: Impact of the anodization time on the photocatalytic activity of TiO2 nanotubes
Source: Beilstein J Nanotechnol. 2018 Oct 4;9:2628–43. doi: 10.3762/bjnano.9.244 (PMC6204776; doi:10.3762/bjnano.9.244)
Supplement: File 1 — Additional experimental data. [file Beilstein_J_Nanotechnol-09-2628-s001.pdf]

Supporting Information

for

Impact of the anodization time on the photocatalytic activity of TiO_2 nanotubes

Jesús A. Díaz-Real*[1,2,3,4], Geyla C. Dubed-Bandomo[1], Juan Galindo-de-la-Rosa[1], Luis G. Arriaga[1], Janet Ledesma-García[3] and Nicolas Alonso-Vante[2]

Address: [1]Centro de Investigación y Desarrollo Tecnológico en Electroquímica, 76703, Querétaro, México; [2]IC2MP, UMR-CNRS 7285, Université de Poitiers, 4 rue Michel Brunet, F-86073 Poitiers, France; [3]Facultad de Ingeniería, División de Investigación y Posgrado, Universidad Autónoma de Querétaro, Centro Universitario Cerro de las Campanas, Querétaro, Qro., C.P. 76010, Mexico and [4]The University of British Columbia, Clean Energy Research Centre, 6250 Applied Science Lane, Vancouver, British Columbia, Canada, V6T 1Z4

Email: Jesús A. Díaz-Real* - jadiaz@mail.ubc.ca

* Corresponding author

Additional experimental data

Dependence of chemical, structural, photoelectrochemical and opto-electronic properties on anodization time

Several of the properties measured and discussed in the main manuscript are plotted as a function of the anodization time. For the F content the integrated area of the F 1s XPS signal was used.

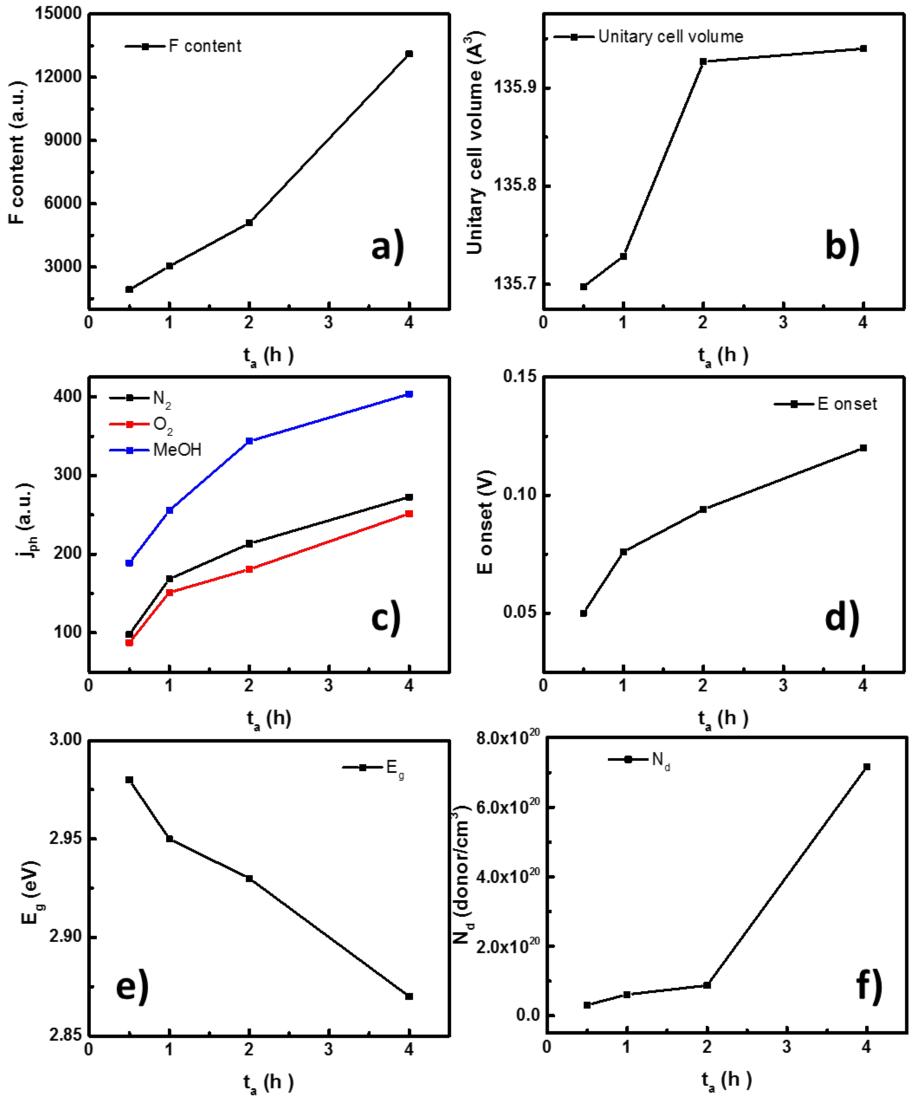

Figure S1: a) F content, b) unitary cell volume, c) photo-generated current density, d) onset potential, e) band-gap, and f) donor density as functions of the anodization time.

Raman spectroscopy

Raman spectra were recorded for samples used as reference to cancel out instrumental artifacts overlying the features observed at 501 and 660 cm^{-1} in the TNT samples. A short anodization (60 s) was performed using the same setup as described in the experimental section and thermally treated using the same procedure. A Degussa P25 sample was also measured for comparison purposes.

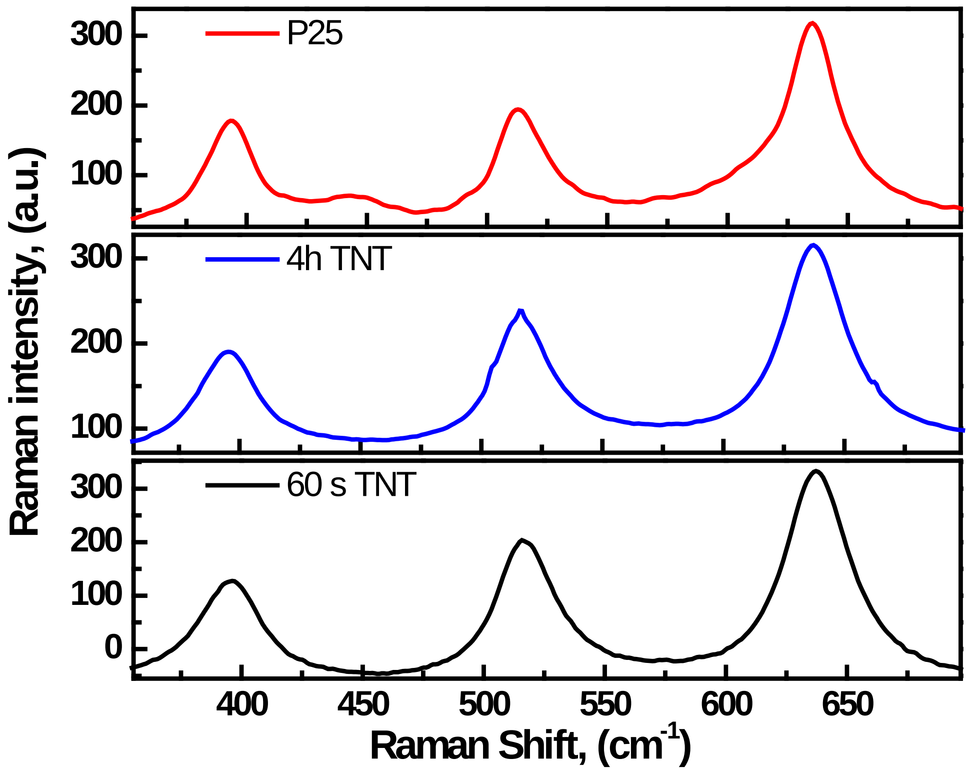

Figure S2: Raman spectra for TNTs grown with different t_a and a Degussa P25 sample. Laser intensity: 2.5 mW/cm^2.

Dependence of photoelectrochemical and opto-electronic properties on the F content

Similar to Figure S1, several measured properties are plotted as functions of the F content in the TNT (understood as the integrated area from the F 1s XPS peak).

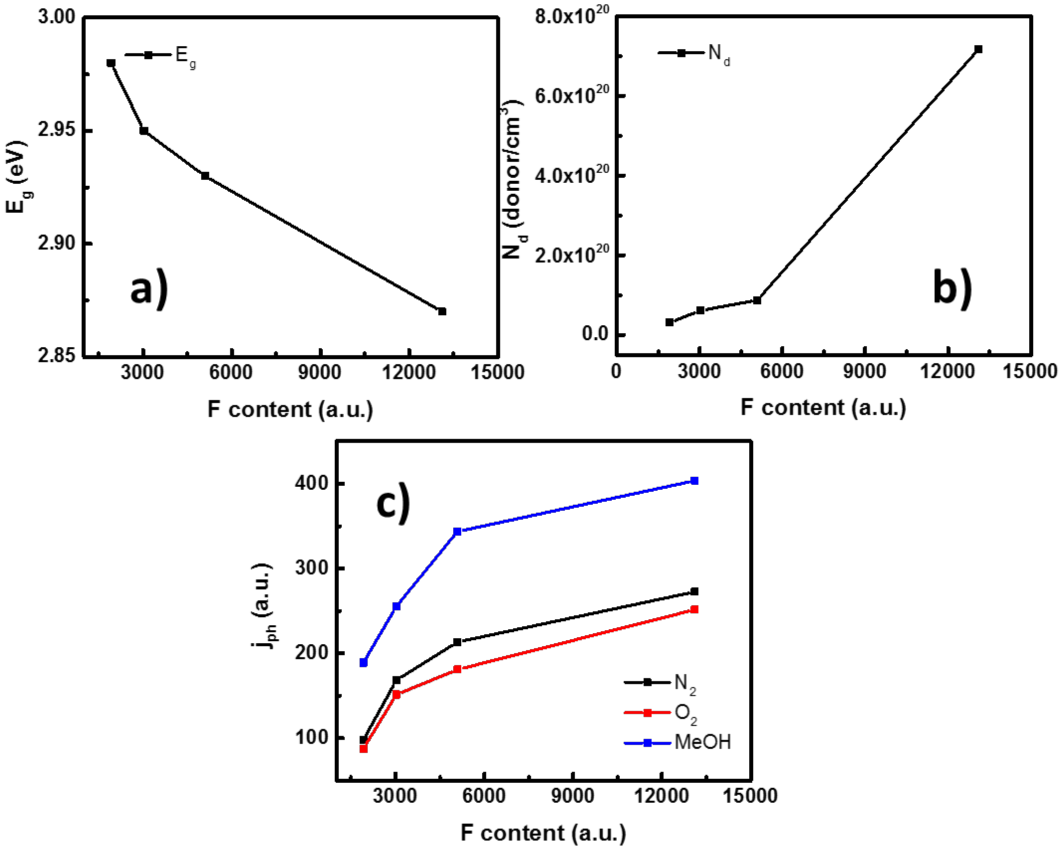

Figure S3: a) Bandgap energy, b) donor density, and c) photo-generated current density as functions of the F content.

Electrochemical measurements of the TNT electrodes during the MB electrooxidation.

Figure S4 shows the cyclic voltammograms of the TNT electrodes in the absence and presence of methylene blue (MB) in darkness and under illumination. Figure S5 shows the electrical charge integrated during the degradation of methylene blue.

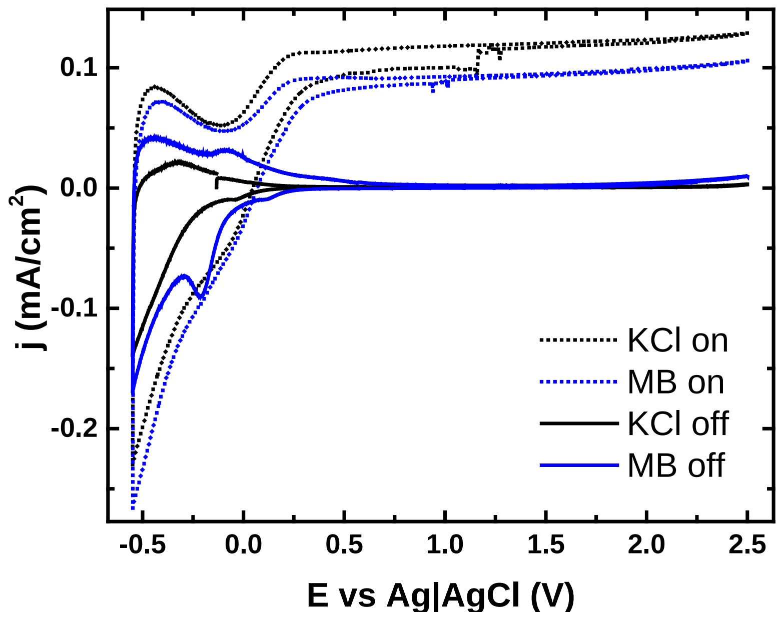

Figure S4: Cyclic voltammograms at v = 50 mV/s TNT (t_a = 0.5 h), in 0.5 M KCl (black lines) and with 20 ppm of methylene blue (blue lines), in darkness (off) and under illumination (on, I_0 = 22.4 mW/cm^2).

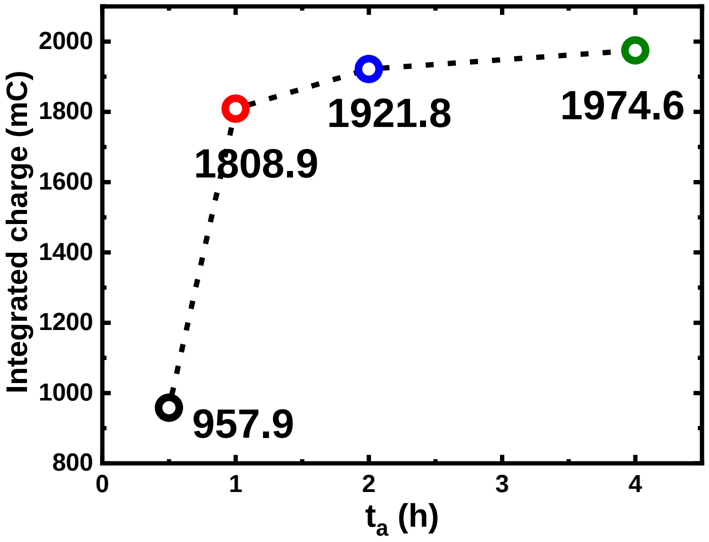

Figure S5: Electrical charge integrated during the degradation of methylene blue after 180 min as a function of the anodization time.
